# Dietary Diversity Feeding Practice and Its Associated Factors among Infants and Young Children Aged between 6 and 23 Months in Birbir Town, Southern Ethiopia

**DOI:** 10.1155/2021/3806360

**Published:** 2021-04-05

**Authors:** Tseganesh Habtamu, Sindu Debebe, Tegegn Solomon, Eshetu Zerihun Tariku, Selamawit Gebeyehu Tiruneh

**Affiliations:** School of Public Health, College of Medicine and Health Sciences, Arba Minch University, Arba Minch, Ethiopia

## Abstract

A significant proportion of infant and young child mortality can be prevented by the provision of a diverse diet. Globally, an estimated 45% of deaths of children under the age of 5 are because of malnutrition. More than two-thirds of these deaths are associated with inappropriate child-feeding practices. This situation is the worst in Ethiopia. Thus, the objective of this study was to determine the status of dietary diversity and identify relevant variables. A community-based cross-sectional study was conducted with 335 randomly selected mothers/caregivers who had infants and young children aged between 6 and 23 months. Data were collected by interview with a structured questionnaire. The data were analyzed using SPSS Version 23. A multivariable logistic regression model was fitted to identify factors associated with dietary diversity practices and statistical significance was declared at *p* < 0.05. Only 12.6% (95% CI; 9.2, 16.6) of children aged 6 to 23 months had adequate dietary diversity. Having a backyard garden and primary education were associated with adequate dietary diversity practices. In this study, the status of adequate dietary diversity feeding practice is low. Therefore, interventions targeting dietary diversity should encourage households to have backyard gardens, and strengthening counseling of mothers/caregivers attending antenatal care and postnatal care is proposed for achieving adequate dietary diversity practices.

## 1. Introduction

Nutrition is a crucial, universally recognized component of children's rights. Children have the right to adequate nutrition and access to safe and nutritious food [1]. However, globally it is estimated that undernutrition is responsible, directly or indirectly, for at least 35% of deaths in children less than 5 years of age. It is also a major cause of disability, preventing children who survive from reaching their full development potential [2].

Based on the evidence that malnutrition is a serious global problem with devastating consequences, governments around the world, with the support of the international community and other concerned parties, are taking seriously their commitments to protect and promote the health and nutritional well-being of infants and young children and set a target to end all forms of malnutrition by 2030 [1, 3]. Also, the importance of better nutrition is recognized under Sustainable Development Goal (SDG2) as “ending hunger, achieve food security and improved nutrition and promote sustainable agriculture goal” and nutrition is an essential component for achieving many of the other SDGs [4].

Infant and young child feeding (IYCF) is one of the actions being implemented as part of the priority child survival and development programs of the United Nations International Children's Emergency Fund (UNICEF) and World Health Organization (WHO). The WHO and UNICEF's recommended different optimal infant and young child-feeding practices, nutritionally adequate (diverse food), and safe complementary feeding are among them [1, 5]. Dietary diversity is a proxy for adequate micronutrient density of foods and it is one of the core indicators for assessing the quality of children's diets [6].

Evidence suggests that a diversified diet has the single greatest potential impact on child survival. A diverse, frequent, and acceptable diet from 6 to 23 months is essential to improved growth, micronutrient status, and cognitive performance, and to prevent wasting. If appropriate complementary feeding practices were scaled up to nearly universal levels, approximately 100,000 deaths in children under 5 years of age could be averted each year [5, 7].

Access to a diverse diet is not a reality for many children in the world. Globally, less than one-third of the world's infants and young children are fed at least four food groups, leaving nearly 70% at risk of undernutrition including micronutrient deficiencies such as vitamin A deficiency, as well as anemia [7]. Research from the Republic of Benin has shown that the magnitude of minimum dietary diversity ranges from 49% among 6–1-month-old to 75% for 18–23-month-old children [8]. Similarly, the Demographic and Health Surveys' result showed 23% of children in Rwanda and 16% in Burundi had minimum dietary diversity [9]. In Ethiopia, only 14% of children 2–5 years of age receive an adequately diverse diet, demonstrating low dietary diversity [10].

Studies have elicited a wide range of predictors of dietary diversity, including the level of maternal education and socioeconomic status of the household [11], households that grow fruit and vegetables and own livestock, age of the child, mothers/caregivers who received IYCF messages during postnatal care (PNC), maternal knowledge on IYCF [12, 13], maternal age, husband's education, marital status, seasonal availability of food, access to all food groups, and source of health information [13].

The government of Ethiopia has demonstrated its commitment to nutrition by developing a standalone National Nutrition Strategy (NNS) [14] and a National Nutrition Program (NNP) [15, 16]. Despite the achievements in establishing strategies and policies, the figures of inadequate dietary diversity show the problem of an unfinished agenda [5] and are among the unachieved targets on the National Nutrition Programme I (NNPI) in Ethiopia [16].

The NNP of Ethiopia supports operational research to answer key operational questions such as why inadequate dietary diversity remains a problem in the country [15]. Thus, it is crucial to have up-to-date information about the existing dietary diversity feeding practice and related variables to create effective complementary feeding interventions. To the investigators' knowledge, the status of dietary diversity feeding practice and its associated factors have not been effectively assessed in the study area. The purpose of this study was, therefore, to determine the magnitude of adequate dietary diversity practices and identify associated variables.

## 2. Materials and Methods

### 2.1. Study Design and Setting

The study was conducted in Birbir town, located in the Gamo and Gofa zones, Ethiopia. Birbir town, the capital of Mirab Abaya district, is located about 465 kilometers from Addis Ababa to the south, at an elevation of 1285 meters above sea level. A community-based cross-sectional study design was conducted from April 26 to May 6, 2018.

### 2.2. Population and Inclusion and Exclusion Criteria

All mothers/caregivers having an infant and young child aged 6 months to 23 months years old in Birbir town were the source population. Mothers/caregivers-child pairs in the selected kebeles (the smallest administrative unit of Ethiopia) during the study period were the study population. All mothers/caregivers-child pair who are permanent residents were included.

### 2.3. Sample Size Determination and Sampling Procedure

A single population proportion formula was used to determine the required sample size based on the following assumptions. The proportion of adequate dietary diversity practice was assumed to be 27.3% [17], with a 95% level of confidence and 5% margins of error, and

(1) $$\begin{array}{c} n=\frac{z_{\alpha /2}^{2}*p\left(1-p\right)}{d^{2}}\\ =\frac{\left(1.96\right)2\times \left(0.273\right)\times \left(0.733\right)}{\left(0.05\right)2}\\ =304.8\\ =305. \end{array}$$

Allowing for a 10% nonresponse rate, the final target sample size was 335.

From the total of eight kebeles in Birbir town, four kebeles (Alpha, Tena Tabya, Menaheria, and Enkutatash Kebeles) were selected randomly by using the lottery method. The total sample size was allocated proportionally based on the number of households who have children between 6 and 23 months of age in their respective kebeles. A list of households was obtained from health extension workers. Systematic random sampling was employed to select 335 study participants. The sampling interval was calculated by dividing the total number of households by the number of households to be selected from each kebele. A random starting point was fixed for each kebele to select the first household by using each kebele center as a starting point and a pen was pinned to identify the beginning direction. Then, all *k*_th_ [4] households were selected until the required sample size was obtained from each of the selected kebeles. When there was more than one eligible study subject in the household, one study subject was selected randomly by the lottery method. When there was no eligible subject in the selected household, the next immediate neighbor household with eligible study subjects was included in the study.

### 2.4. Data Collection Tool and Procedure

Data were collected using interviewer-administered structured-questionnaires. The questionnaire was developed by reviewing related literature [17–23]. It was initially prepared in English and then translated into Amharic and backtranslated to English to ensure its consistency. Then, it was pretested in Arba Minch town on 5% of the sample size (*N* = 17). The questionnaire included sociodemographic and economic variables, infant and young child characteristics, household characteristics, maternal and child health-service utilization characteristics, maternal awareness about dietary diversification, external influences to the mothers/caregivers on child-feeding, and dietary diversification practices. Dietary diversity practiced was assessed with nine questions. The seven food groups used for tabulation of this indicator were cereals, roots, and tubers; legumes and nuts; dairy products (milk, yogurt, and cheese); flesh foods (meat, fish, poultry, and liver/organ meats); eggs; vitamin A rich fruits and vegetables; and other fruits and vegetables [2, 16].

### 2.5. Data Quality Management

The questionnaire was pretested and modifications were made with regard to skipping and flow of the questions. Daily supervision was carried out to check the completeness of the questionnaire. Errors identified during data collection were corrected at the field and errors that occurred during/after data entry were addressed by revising the original questionnaire.

### 2.6. Data Processing and Analysis

Data were coded and entered into EpiData version 3.1 and exported to SPSS version 23 for analysis. First, descriptive analyses were conducted to describe the variables involved in the study. Then, the magnitude of dietary diversity was computed. Bivariate logistic regression was conducted to assess the association between each categorical variable with the outcome variable and to identify candidate variables (with a *p* value ≤0.25) to be included in the multivariable logistic regression model. Then, the multivariable logistic regression model was fitted using the default (enter) variable selection method to identify variables independently associated with the outcome variable of interest at a 5% significance level. The adjusted odds ratio (AOR) and the corresponding 95% CI for the variables in the final model were reported. The goodness of fit of the final model was assessed using Hosmer and Lemeshow goodness of fit test. The model was a good fit with a *p* value of 0.3. The over percentage of classification was 87.4%.

### 2.7. Operational Definitions

Dietary diversity was defined as adequate if children (aged 6–23 months) received foods from at least four of seven food groups ((1) Grains, roots, and tubers, (2) legumes and nuts, (3) dairy products, (4) flesh foods, (5) eggs, (6) vitamin A rich fruits and vegetables, (7) other fruits and vegetables) within the 24 h preceding the interview.

### 2.8. Ethical Consideration

The official letter from the School of Public Health, Arba Minch University, was secured and a permission letter was obtained from Gamo Zonal Health Department. Participants were informed about the objectives, risks, and benefits of the study. Verbal consent was obtained. Participant's involvement in the study was voluntary and those who wish to quit their participation at any stage were informed to do so without any restriction.

## 3. Results

### 3.1. Sociodemographic, Economic, and Household Characteristics

A total of 334 mothers/caregiver-child pairs participated in this study to create a response rate of 99.7%. One hundred sixty-three (48.8%) of mothers/caregivers were less than or equal to 24 years of age. Of the total participants, 160 (47.9%) were orthodox in religion. Most of the participants, 134 (40.1%), had completed primary education (grades 1–8) and 160 (47.9%) of the women were housewives (Table 1).

### 3.2. Infant and Young Child Health-Related Characteristics

Most of the children (*n* = 202, 60.5%) were between 12 and 23 months of age, and more than half (*n* = 184, 55.1%) were male. From the total number of children who participated in the study, 317 (94.9%) were being breastfed at the time of the survey. Most of the children (*n* = 244, 73.1%) were not, or had not been, ill in 2 weeks before the survey (Table 2).

### 3.3. Maternal Health-Service Utilization

A total of 324 participants (97%) had antenatal care follow-up when they were pregnant for the index child, and 302 (90.4%) of the 334 mothers/caregivers delivered their children at health facilities. Regarding postnatal care visits, from 230 mothers/caregivers who had visits, 140 (60.9%) had three or more visits (Table 3).

### 3.4. Child-Feeding Practices

A total of 308 (92.2%) participants did not provide varieties of food for their children. Among the participants who had been influenced by the diversification of food, 16 were influenced by neighbors and family members.

**Figure 1:**
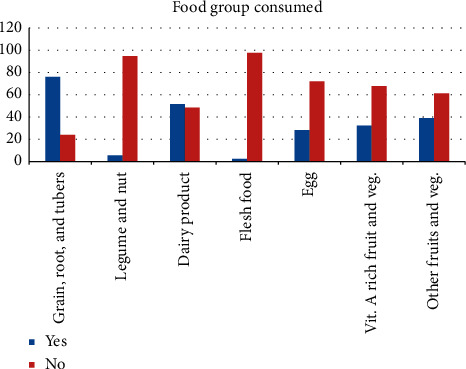
Types of food groups consumed among 6–23 months children. Factors associated with appropriate dietary diversity feeding practice.

The proportion of children who were receiving the recommended dietary diversity was 12.6% (95% CI; 9.2, 16.6). Most of the children (*n* = 254, 76.0%) feed on grain, roots, and tubers, followed by dairy products 172 (51.5%); only a few (*n* = 18, 5.4%) consumed legumes and nuts (Figure 1).

In the multivariable analysis, higher maternal educational status and having a backyard garden were significantly associated with appropriate dietary diversity practice.

Children from households that had backyard gardens had higher odds of maintaining adequate dietary diversity when compared with children from households that did not have backyard gardens (AOR = 2.34; 95% CI: 1.11, 5.10). Furthermore, the odds of feeding adequate dietary diversity to a child's age of 6–23 months were lower among mothers/caregivers who had attained only primary education (AOR = 0.30; 95% CI: 0.12, 0.81) compared with those who had no formal education (Tables 4 and 5).

## 4. Discussion

The key contribution of this paper is to show the status of dietary diversity practices and associated variables among 6–23-month children in Birbir town. The findings have important implications, particularly in Ethiopia, where the burden of malnutrition especially micronutrient deficiency is high and dietary diversity is a proxy indicator for adequate micronutrient density of foods [24].

The results in the current study are comparable with the EDHS 2016 report in which only 14% of children had an adequately diverse diet [10]. This result is consistent with previous studies conducted in the country in which dietary diversity feeding practice is found to be (12.6%) in Dangila town [25] and (10.6%) Gorche districts of Sidama zone [12].

The Ethiopian government on the national nutritional program had set a target to increase the proportion of children aged 6–23 months with adequate dietary diversity score to be 20% by 2015 [16] and 40% by the end of 2020 [15]. However, in this study, the status of dietary diversity practice was found to be 12.6% (95% CI: 9.2, 16.6) which is very low compared with these targets. Similarly, the dietary diversity feeding practice observed in the present study was low compared with previous studies conducted in Ethiopia (Addis Ababa (59.9%) [26], Haramaya town (25.2%) [27], Wolita sodo town (27.3%) [17], and Bale Zone (28.5%) [20]. It is also lower than the study from Moramanga district (42.1%) and Morondava district (47.6%) at Madagascar [11]. The discrepancy with the study done in Moramanga and Morondava might be due to the difference in the age of the participants. The previous study measures dietary diversity of children from 6 to 59 months but in this study dietary diversity was assessed among 6 to 23-month-old children. As the age of children increases, it may encourage mothers/caregivers to initiate complementary feeding which increases the probability of providing diversified foods [28]. Furthermore, the difference with the studies done at Oromia region (Bale Zone and Haramaya Town) [20, 27] might be due to different interventions to improve proper child-feeding practices in the local contexts. A possible reason for dissimilarity with the study done at Wolita sodo might be the sample size. The sample size of this study was lower than the sample size of the study done at Wolita sodo.

In this study, being a child in a household that had a backyard garden was an important determinant of adequate dietary diversity feeding practice, suggesting the importance of interventions at the household level. This is consistent with another study conducted before [12]. This may be explained by the fact that having a backyard garden can provide an entry point to reach mothers/caregivers in households and influence their feeding practices by improving access to local foods [5, 29].

We found a negative association between maternal educational status and dietary diversity feeding practices. Mothers/caregivers who had primary education were found to be less likely to practice adequate dietary diversity feeding compared with those who have no formal education. This result is contradictory to that of previous studies done [20, 25–27]. This discrepancy might be explained by the maternal experience of ANC and PNC visits. In this study, 90% and 70% of mothers/caregivers who had no formal education attend at least four and above ANC visits and PNC visits, respectively, whereas 67.5% and 62.5% of mothers/caregivers who had primary education attend at least 4 and above ANC visits and PNC visits, respectively. As a result, mothers/caregivers who had no formal education might have high opportunities for getting health and nutrition knowledge by trained health workers on IYCF practices which helped them to feed diverse diets to their children [20].

To sum up, the study has incorporated many variables and successfully showed important recommendations that can be used in the formulation of interventions to improve IYCF practice in the study area. However, this study may have its limitations in that it used only a 24 h recall method which tells us only a one-time phenomenon but did not demonstrate the dietary habits of the participants and affected by the variation of days. Also, there might be social desirability and recall bias in reporting the type of food given to children. However, an effort was taken to minimize such biases by probing the study participants and explaining the purpose for asking particular questions.

## 5. Conclusions and Recommendation

In this study, the status of adequate dietary diversity feeding practice is low. Having a backyard garden and primary education were independent factors associated with adequate dietary diversity. Therefore, interventions targeting dietary diversity should encourage households to have backyard gardening, and strengthening counseling of mothers/caregivers attending ANC and PNC is proposed for achieving adequate dietary diversity feeding practices.

## Figures and Tables

**Table 1:** Sociodemography and economy of mothers/caregivers and household characteristics on the study conducted to assess dietary diversity feeding practice and associated factors among 6–23-month children in Birbir Town, Ethiopia, 2018 (*n* = 334).

Variable		Number	Percent
Age of the mothers/caregivers (in years)	≤24	163	48.8
	25–30	119	35.6
	≥31	52	15.6
	No formal education	74	22.2

Educational status of the mothers/caregivers at the time of the survey	Grades 1–8	134	40.1
	Grades 9–12	86	25.7
	College and above	40	12.0

Current marital status of mothers/caregivers at the time of the survey	Single	8	2.4
	Married	316	94.6
	Divorced/separated	6	1.8
	Widowed	4	1.2

Current occupational status of the mothers/caregivers at the time of the survey	Housewife	160	47.9
	Farmer	17	5.1
	Merchant	68	20.4
	Government employee	59	17.7
	Daily laborer	16	4.8
	Private	4	1.2
	Other	10	3.0

Religion of the mothers/caregivers	Orthodox	160	47.9
	Protestant	154	46.1
	Muslim	20	6.0

Read newspaper, magazine, or any other material	No	50	15.0
	Yes	284	85.0

Do you listen to the radio?	Yes	200	59.9
	No	134	40.1

Do you watch TV?	Yes	244	73.1
	No	90	26.9

Monthly income (in birr ^*∗*^)	≤3000	284	85.0
	>3000	50	15.0

Family size	≤3	104	31.1
	4–6	187	56.0
	≥7	43	12.9

Head of the house	Male	314	94.1
	Female	20	5.9

Number of under-five-year-old children	1–2	306	91.6
	≥3	28	8.4

Mothers/caregivers involved in decisions about feeding child (ren)	Yes	328	98.2
	No	6	1.8

A household with backyard gardening	Yes	92	27.5
	No	242	72.5

A place to obtain food	Own production	242	72.5
	Purchase	92	27.5

^*∗*^Birr: Ethiopia's official currency.

**Table 2:** Health-related characteristics of children (*N* = 334).

Variable		Number	Percent
Age of the child (in months)	6–11	132	39.5
	12–23 months	202	60.5

Sex	Male	184	55.1
	Female	150	44.9

Breastfeeding child at the time of the survey	Yes	317	94.9
	No	17	5.1

Birth order	First	148	44.3
	Second	106	31.7
	Third and above	80	24.0

Birth interval	<24 months	16	8.6
	≥24 months	170	91.4

Morbidity for the last 2 weeks	Yes	90	26.9
	No	244	73.1

Type of morbidity	Diarrhea	28	31.1
	Vomiting	24	26.6
	Fever with chills	20	22.2
	Other	18	20.1

Child growth monitoring follow-up	Yes	262	78.4
	No	72	21.6

Vaccination history	Vaccinated	328	98.2
	Not vaccinated	6	1.8

**Table 3:** Maternal health-service utilization and child-feeding practices.

Variable		Number	Percent
ANC visits	Yes	324	97.0
	No	10	3.0

Number of ANC visits	1 to 3 visits	56	16.8
	Four or more visits	268	80.2

Place of delivery	Health facility	302	90.4
	Other	32	9.6

PNC visits	Yes	230	68.9
	No	104	31.1

Number of PNC visits	One visit	30	13.0
	Two visits	60	26.0
	Three or above visits	140	60.9

Heard when to start complementary food	Yes	306	91.6
	No	28	8.4

Source of information about complementary food	Health professionals	233	76.1
	Mass media	36	11.7
	Family members	23	7.5
	Other	14	4.6

**Table 4:** The result of bivariate and multivariable logistic regression to assess the association of adequate dietary diversity feeding practice and sociodemographic, economic, and household factors among 6–23-month children in Birbir Town, Ethiopia, 2018, (*n* = 334).

Variable	Dietary diversity		COR (95% CI)	AOR (95% CI)
	Adequate	Inadequate		
	*N* (%)	*N* (%)		
Age of the mothers/caregivers (years)				
≤24	20 (12.3)	143 (87.7)	1	
25–30	14 (11.8)	105 (88.2)	0.95 (0.46, 1.97)	
≥31	8 (15.4)	44 (84.6)	1.30 (0.54, 3.16)	

Educational status of mothers/caregivers				
No formal education	14 (18.9)	60 (81.1)	1	1
Primary	10 (7.5)	124 (92.5)	0.35 (0.15, 0.82) ^*∗*^	0.30 (0.12, 0.81) ^*∗∗*^
Secondary	16 (18.6)	70 (81.4)	0.98 (0.44, 2.17)	0.85 (0.35, 2.06)
College and above	2 (5.0)	38 (95.0)	0.23 (0.05, 1.05) ^*∗*^	0.23 (0.05, 1.16)

Marital status at the time of the survey				
Married	38 (12.0)	278 (88.0)	0.48 (0.12, 1.88) ^*∗*^	0.46 (0.12, 1.83)
Other	4 (22.2)	14 (77.8)	1	

Monthly income (in birr) ^*∗*^				
≤3000	36 (12.7)	248 (87.8)	1	
>3000	6 (12.0)	44 (88.0)	0.94 (0.37, 2.36)	

Family size				
≤3	8 (7.7)	96 (92.3)	0.51 (0.16, 1.58) ^*∗*^	0.85 (0.24, 2.99)
4–6	28 (15.3)	159 (85.0)	1.08 (0.42, 2.81)	1.14 (0.39, 3.24)
≥7	6 (14.0)	37 (86.0)	1	1

Read newspaper				
Yes	34 (12.0)	250 (88.0)	0.71 (0.31, 1.64)	
No	8 (16.0)	42 (84.0)	1	

Listen to radio				
Yes	24 (12.0)	176 (88.0)	0.88 (0.45, 1.69)	
No	18 (13.4)	116 (86.6)	1	

Watch TV				
Yes	28 (11.5)	216 (88.5)	0.70 (0.35, 1.41)	
No	14 (15.6)	76 (84.4)	1	

A household with backyard gardening				
Yes	18 (19.6)	74 (80.4)	2.21 (1.14, 4.23) ^*∗*^	2.34 (1.11, 5.10) ^*∗∗*^
No	24 (9.9)	218 (90.1)	1	1

A place to obtain food				
Own production	34 (14.0)	208 (86.0)	1.72 (0.76, 3.86) ^*∗*^	0.87 (0.23, 3.24)
Purchased	8 (8.7)	84 (91.3)	1	1

Head of household				
Male	40 (12.7)	274 (87.3)	1.31 (0.29, 5.87)	
Female	2 (10.0)	18 (90.0)	1	

^*∗*^Significant at *p* value ≤0.25;  ^*∗∗*^significant at *p* value <0.05; COR: crude odds ratio; AOR: adjusted odds ratio,  ^*∗*^Birr**:** Ethiopia's official currency.

**Table 5:** The result of bivariate and multivariable logistic regression to assess the association of adequate dietary diversity feeding practice and child health and maternal health-service utilization related factors among 6–23-month children in Birbir Town, Ethiopia, 2018.

Variable	Dietary diversity		COR (95% CI)	AOR (95% CI)
	Adequate	Inadequate		
	*N* (%)	*N* (%)		
Age of child				
6–11 months	12 (9.1)	120 (90.9)	1	1
≥12 months	30 (14.9)	172 (85.1)	1.74 (0.85, 3.54) ^*∗*^	0.74 (0.34, 1.63)
Sex of child				
Male	22 (12.0)	162 (88.0)	0.88 (0.46, 1.68)	
Female	20 (13.3)	130 (86.7)	1	
Breastfeeding				
Yes	36 (11.4)	281 (88.6)	1	1
No	6 (35.3)	11 (64.7)	4.26 (1.48, 12.21) ^*∗*^	2.98 (0.85, 10.45)
Morbidity for the last two weeks				
Yes	10 (11.1)	80 (88.9)	0.83 (0.38, 1.76)	
No	32 (13.1)	212 (86.9)	1	
Child growth monitoring follow-up				
Yes	36 (13.7)	226 (86.3)	1.75 (0.71, 4.34) ^*∗*^	1.95 (0.59, 6.45)
No	6 (8.3)	66 (91.7)	1	
ANC visits				
≤3 visits	4 (6.1)	62 (93.9)	1	1
4 and above visits	38 (14.2)	230 (85.8)	2.56 (0.88, 7.45) ^*∗*^	1.95 (0.59, 6.45)
PNC visits				
Yes	34 (14.8)	196 (85.2)	2.08 (0.93, 4.67) ^*∗*^	2.18 (0.69, 6.87)
No	8 (7.7)	96 (92.3)	1	1
Place of delivery index child				
Health facility	38 (12.6)	264 (87.4)	1.01 (0.34, 3.03)	
Other	4 (12.5)	28 (87.5)	1	
Heard when to start complementary food				
Yes	38 (12.4)	268 (87.6)	0.85 (0.28, 2.58)	
No	4 (14.3)	24 (85.7)	1	
Influence on child feeding				
Yes	4 (15.4)	22 (84.6)	1.29 (0.42, 3.95)	
No	38 (12.3)	270 (87.7)	1	

Note:  ^*∗*^significant at *p* value ≤0.25;  ^*∗∗*^significant at *p* value <0.05; COR: crude odds ratio; AOR: adjusted odds ratio.

## Data Availability

The data used to support the findings of this study are not publicly available due to restrictions on the publication of human subjects' data but these data can be available from the corresponding author upon reasonable request.

## Abbreviations

ANC: Antenatal care
IYCF: Infant and young child feeding
NNP: National Nutrition Program
NNS: National Nutrition Strategy
PNC: Postnatal care
UNICEF: United Nations International Children's Emergency Fund
WHO: World Health Organization.
